# Host genetics of infectious diseases: old and new approaches converge.

**DOI:** 10.3201/eid0404.980426

**Published:** 1998

**Authors:** A V Hill

**Affiliations:** University of Oxford, United Kingdom.


### Host Genetics of Infectious Diseases: Old and New Approaches Converge

The increasing interest in infectious disease genetics over the last 5 years reflects several trends in the biomedical sciences, not least the explosion of knowledge in human genomics. However, infectious disease genetics is not a new field; some of the largest twin studies were performed more than 50 years ago, and the first malaria resistance gene was identified soon afterwards. The current excitement reflects a recent increase in power in several of the approaches being used to map and identify the genes responsible for variable susceptibility to many major infectious diseases. Four distinct approaches have been used to identify infectious disease susceptibility and resistance genes; these approaches may now be converging.

### Approaches

### Candidate Genes

The most popular type of study in the host genetics of infectious disease compares the allele or genotype frequencies of so-called "candidate genes" in clinical cases with matched controls. Candidate genes are selected on the basis of their function and likely relevance to the disease of interest and on the possession of one or more genetic variants (1). This approach has been very successful. Starting with the globin genes and glucose-6-phosphate deficiency, several candidate genes were found in case-control studies to be associated with malaria resistance. A different approach associated the Duffy blood group with *Plasmodium vivax* malaria resistance many years before the blood group was known to be a chemokine receptor. In many infectious diseases, HLA variation has been studied, and several associations have been established. The early associations between HLA-DR2 and susceptibility to leprosy and tuberculosis (TB) in India have been reconfirmed in recent years. Most recently, chemokine receptors and a deletion variant of CCR5 have been found to affect both risk for HIV-1 infection and rate of progression to AIDS.

Despite these successes, problems remain with the candidate gene approach. Most sample sizes have been adequate to detect only very strong associations; however, less marked associations may be more common. Matching controls to cases may be problematic and has led to the recent trend for family-based association studies in other types of polygenic disease. Frequent geographic heterogeneity in HLA associations has complicated the interpretation of studies of these important genes. Finally, the limited effects observed in most studies suggest that this approach might be missing some other genes with more major effects. Nonetheless, with more promising candidate polymorphisms appearing each month, the popularity of this approach will likely increase.

### Mouse Genetics

A quite different approach to finding human infectious disease resistance and susceptibility genes has been identifying relevant murine genes. The relative ease of mapping susceptibility genes in inbred strains of mice has led to the mapping of numerous named loci in mice by linkage analysis. In some cases, such as with the murine Mx influenza resistance locus, the gene has actually been identified (2). Far more genes have been mapped in this way than have been identified. A well-studied exception is the murine *Nramp1* gene that was positionally cloned on chromosome 1 (3) after this locus was shown to influence susceptibility to certain leishmanial, mycobacterial and salmonella infections in mice. Another important recent approach has been the use of gene knockout mice to determine whether particular genes affect susceptibility to various infectious diseases.

A difficulty with the mouse genetics approach is that a gene identified in mouse studies may turn out to lack polymorphism in humans. This difficulty is particularly relevant for artificially generated knockout mice; inactivating mutations of key genes may be too deleterious to humans. Such mutations, like mutations of the interferon-gamma receptor gene (4), may, however, turn up rarely in immunodeficient children. However, even mutations that are polymorphic in inbred mouse strains, such as the *Nramp1* variant, may be nonpolymorphic in wild mouse populations. Given the differences in selection pressure that mice and humans have been exposed to over tens of millions of years, the major susceptibility genes in the two species are unlikely to be the same. Another difficulty is, of course, the extent to which a murine model may mimic human infectious disease. The human malarias, for example, cannot be studied in mice.

### Complex Segregation Analysis

Complex segregation analysis models the transmission of the disease in multicase families and compares the likelihood of different ways of inheriting the disease. Several parameters may be optimized, including the contribution of a potential single major gene, polygenic and environmental components, allele frequencies, and dominance. The remarkable result is the regularity with which a single major gene is reported. For example, a single major gene has thus been implicated in leprosy, schistosomiasis, TB, and even malaria (regulating parasite densities) (5). Even allowing for some publication bias, these remarkable results are surprising in view of the highly polygenic nature of infectious disease susceptibility suggested by candidate gene analysis. A major gene, as proposed in these models, would need both a high frequency and very high odds ratio to account for a substantial proportion of the overall genetic effect. One explanation might be that such single major genes remain to be identified in these and perhaps many other infectious diseases.

The usefulness of complex segregation analysis is difficult to assess because in no case has the single major gene proposal been confirmed by mapping and identifying such a gene. However, this difficulty may be ending as genome scanning technology is increasingly applied to infectious diseases.

### Human Genomewide Analysis

The most recent approach applied to human infectious diseases is mapping and subsequently identifying major genes affecting susceptibility or resistance through genomewide scans. This approach entails an initial linkage analysis in large numbers of multicase families, which is followed by association study analysis for gene identification. Typically, a few hundred microsatellite markers search for evidence of increased sharing of parental alleles identical by descent in affected sibling pairs. This nonparametric approach does not require information on how the disease was inherited, which is almost always unknown. However, in schistosomiasis the results of complex segregation analysis have been used in a parametric, or model-based, analysis to map a susceptibility gene to chromosome 5 in a small number of Brazilian families (6), as reviewed by Abel and Dessein in this issue. The degree to which this genomewide approach can be used to map major susceptibility genes in reasonable numbers of families remains uncertain. However, the genes with the largest effects are those that can be mapped with most power and may be of greatest interest.

The main limitation of a genomewide linkage scan in polygenic infectious diseases is that its power is lower than that of association studies. Recruiting the required large numbers of clinically well-defined multicase families may require multicenter collaborations. Technical advances may make genomewide association studies feasible, which would remove the need for the use of multicase families, although the requirement for large numbers of cases would remain.

### Approach Convergence

Until recently, these various genetic approaches were typically pursued by different research groups with relatively little synergistic interaction. The major candidate genes studied in humans were different from those mapped in mice, and none of the associations found appeared strong enough to fit the single major genes being proposed by complex segregation analysis. However, researchers working on the *Nramp1* gene in mice suggested that the human homologue, *NRAMP1*, could correspond to a major mycobacterial susceptibility gene suggested by segregation analysis in humans. This theory has now become testable with the identification of numerous polymorphisms in *NRAMP1*. A clear association emerges between variation in this gene and TB susceptibility, at least in a West African population (7). Thus a gene identified through whole genome analysis in mice, potentially the major gene suggested by complex segregation analysis, is associated with susceptibility when investigated as a candidate gene in a human case-control study. The separate approaches have converged, we hope for the first of many meetings.

However, little in complex disease genetics is simple. The effect observed in Gambia is different from BCG susceptibility in mice in that in human TB, susceptibility appears dominant (7), while in mice it is recessive. Also, the effect in humans, though highly statistically significant, is relatively modest, perhaps a few percentage points of the overall genetic component of susceptibility. Although not the elusive major gene, this effect is well worth investigating for the light it may throw on mechanisms of disease susceptibility. As other candidate genes are identified from mouse studies, they may add to the long list of candidate genes available for human association studies. Meanwhile, the hunt for elusive major susceptibility genes continues. Ongoing genome scans should soon help determine whether these genes really exist in particular infectious diseases; if they do, their mechanisms of action as well as the forces underlying their evolutionary maintenance should be of great interest.

**Adrian V.S. Hill**
University of Oxford, Oxford, United Kingdom

### References

1. Hill AVS. The immunogenetics of resistance to human infectious diseases. Ann Rev Immunol. In press 1998.
2. Staeheli P, Haller O, Boll W, Lindenmann J, Weissmann C. Mx protein: constitutive expression in 3T3 cells transformed with cloned Mx cDNA confers selective resistance to influenza virus. Cell 1986;44:147-58.
3. Vidal SM, Malo D, Vogan K, Skamene E, Gros P. Natural resistance to infection with intracellular parasites: isolation of a candidate for Bcg. Cell 1993;73:469-85.
4. Newport MJ, Huxley CM, Huston S, Hawrylowicz CM, Oostra BA, Williamson R, et al. A mutation in the interferon-gamma-receptor gene and susceptibility to mycobacterial infection. N Engl J Med 1996;335:1941-9.
5. Abel L, Cot M, Mulder L, Carnevale P, Feingold J. Segregation analysis detects a major gene controlling blood infection levels in human malaria. Am J Hum Genet 1992;50:1308-17.
6. Marquet S, Abel L, Hillaire D, Dessein H, Kalil J, Feingold J, et al. Genetic localization of a locus controlling the intensity of infection by *Schistosoma mansoni* on chromosome 5q31-q33. Nat Genet 1996;14:181-4.
7. Bellamy R, Ruwende C, Corrah T, McAdam KJ, Whittle HC, Hill AVS. Variation in the NRAMP1 gene and susceptibility to tuberculosis in West Africans. N Engl J Med 1998;338:640-4.

## References

[PDF_00242] Abel L., Cot M., Mulder L., Carnevale P., Feingold J. (1992). Segregation analysis detects a major gene controlling blood infection levels in human malaria.. Am J Hum Genet.

[PDF_00251] Bellamy R., Ruwende C., Corrah T., McAdam K. P., Whittle H. C., Hill A. V. (1998). Variations in the NRAMP1 gene and susceptibility to tuberculosis in West Africans.. N Engl J Med.

[PDF_00246] Marquet S., Abel L., Hillaire D., Dessein H., Kalil J., Feingold J., Weissenbach J., Dessein A. J. (1996). Genetic localization of a locus controlling the intensity of infection by Schistosoma mansoni on chromosome 5q31-q33.. Nat Genet.

[PDF_00238] Newport M. J., Huxley C. M., Huston S., Hawrylowicz C. M., Oostra B. A., Williamson R., Levin M. (1996). A mutation in the interferon-gamma-receptor gene and susceptibility to mycobacterial infection.. N Engl J Med.

[PDF_00229] Staeheli P., Haller O., Boll W., Lindenmann J., Weissmann C. (1986). Mx protein: constitutive expression in 3T3 cells transformed with cloned Mx cDNA confers selective resistance to influenza virus.. Cell.

[PDF_00234] Vidal S. M., Malo D., Vogan K., Skamene E., Gros P. (1993). Natural resistance to infection with intracellular parasites: isolation of a candidate for Bcg.. Cell.

